# Identifying Scoliosis in Population-Based Cohorts: Automation of a Validated Method Based on Total Body Dual Energy X-ray Absorptiometry Scans

**DOI:** 10.1007/s00223-019-00651-9

**Published:** 2020-01-09

**Authors:** Amir Jamaludin, Jeremy Fairbank, Ian Harding, Timor Kadir, Tim J. Peters, Andrew Zisserman, Emma M. Clark

**Affiliations:** 1 Department of Engineering Science, University of Oxford, Oxford, UK; 2 Nuffield Department of Orthopaedics, Rheumatology and Musculoskeletal Science, University of Oxford, Oxford, UK; 3 North Bristol NHS Trust, Bristol, UK; 4 Optellum Ltd, Oxford, UK; 5 Musculoskeletal Research Unit, Bristol Medical School, University of Bristol, Bristol, UK

**Keywords:** Scoliosis, ALSPAC, Bristol DXA scoliosis method, Machine learning

## Abstract

Scoliosis is a 3D-torsional rotation of the spine, but risk factors for initiation and progression are little understood. Research is hampered by lack of population-based research since radiographs cannot be performed on entire populations due to the relatively high levels of ionising radiation. Hence we have developed and validated a manual method for identifying scoliosis from total body dual energy X-ray absorptiometry (DXA) scans for research purposes. However, to allow full utilisation of population-based research cohorts, this needs to be automated. The purpose of this study was therefore to automate the identification of spinal curvature from total body DXA scans using machine learning techniques. To validate the automation, we assessed: (1) sensitivity, specificity and area under the receiver operator curve value (AUC) by comparison with 12,000 manually annotated images; (2) reliability by rerunning the automation on a subset of DXA scans repeated 2–6 weeks apart and calculating the kappa statistic; (3) validity by applying the automation to 5000 non-annotated images to assess associations with epidemiological variables. The final automated model had a sensitivity of 86.5%, specificity of 96.9% and an AUC of 0.80 (95%CI 0.74–0.87). There was almost perfect agreement of identification of those with scoliosis (kappa 0.90). Those with scoliosis identified by the automated model showed similar associations with gender, ethnicity, socioeconomic status, BMI and lean mass to previous literature. In conclusion, we have developed an accurate and valid automated method for identifying and quantifying spinal curvature from total body DXA scans.

## Introduction

Scoliosis is defined as lateral curvature of the spine ≥ 10°, as measured using the Cobb method on a standing spinal radiograph [1]. The most common form is adolescent-onset idiopathic scoliosis (AIS), defined as occurring between age 10 years and skeletal maturity [2]. It is not always a benign structural abnormality, although the mortality rate for individuals with AIS is comparable to that of the general population [3]. Severe AIS may result in early degenerative joint disease [4], negative body image [5] and psychosocial disturbances [6]. Even small spinal curves in adolescents, which may not have presented to spinal units, are associated with an increased risk of future back pain and time off school [7].

However, our understanding of the causes of curve initiation and progression is hampered by lack of prospective population-based studies, driven mainly by the serious ethical concerns over performing spinal radiographs in healthy populations because of the radiation exposure, equivalent to an entire year’s background radiation [8].

To address this, we have validated a manual method for measuring spinal curvature using total body dual energy X-ray absorptiometry (DXA) scans for research purposes [9]: the DXA Scoliosis Method (DSM). As previously published [9], the manual DSM is reliable (substantial agreement was seen with a kappa of 0.75), repeatable (95% of repeat measures were within 5°, and there was no change in interobserver variability as curve size increased) and accurate (comparison with the gold standard of using the Cobb method on standing spinal radiographs was as expected). The manual DSM also produced valid estimates of prevalence of scoliosis, with expected gender ratio [9].

This has allowed us to start to identify predictors of AIS onset utilising population-based cohorts that have already performed DXA scans for previous research into determinants of bone density. The DSM has been applied to participants in the Avon Longitudinal Study of Parents and Children (ALSPAC) at age 9 years (*n* = 7000) and age 15 years (*n* = 5000) and results have shown we can identify altered body composition [10] and reduced physical activity [11] in children, prior to onset of their spinal curve. Interestingly, reduced physical ability is seen as early as age 18 months in those who go on to develop AIS between ages 9 and 15 [11]. This suggests that clinical features other than characteristics of the spinal deformity itself may indeed be useful prognostic indicators.

The main goal of further epidemiological analysis of scoliosis is to identify predictors of spinal curve progression. This would allow generation of a clinical prediction tool to identify people at low risk of curve progression, for example, who would then require less rigorous monitoring. However, even though the prevalence of AIS is relatively common (5.9% at age 15 [9]), ALSPAC is not large enough on its own, and to carry out appropriately powered epidemiological studies we need to combine data from multiple research cohorts. We have identified additional research cohorts that already have total body DXA scans already performed (approximately 84,000 scans). However, application of our manual DSM on all relevant DXA images is unfeasible in terms of time and cost. For example, the original annotation 12,000 ALSPAC images required 200 staff days of analysis time.

Therefore the aim of this work was to develop and validate a fully automated version of the manual DSM method using a machine learning approach. The intended purpose of this automated method is to exploit population-based cohorts for research purposes. The availability of large datasets and increasingly powerful computational resources has made the development of such techniques feasible with applications ranging from fibrotic lung disease [12] to ophthalmology [13]. The scoliosis automation proposed here is based on the ideas developed in the SpineNet software [14], a deep-learning based automated tool for quantitative assessment of spinal degeneration on lumbar MRI imaging studies.

## Methods

### Study Population

ALSPAC is a geographically-based UK cohort that recruited pregnant women residing in Avon (South-west England), with an expected date of delivery between 1 April 1991 and 31 December 1992 [15, 16]. A total of 14541 pregnancies were enrolled, with 14062 children born; see www.alspac.bris.ac.uk for more information. The study website contains details of all the data that are available through a fully searchable data dictionary and variable search tool available at https://www.bris.ac.uk/alspac/researchers/our-data/. This study is based on 7298 children who had DXA scans at the aged 9 research clinic, 5122 who had DXA scans at the aged 15 research clinic, and 4969 who had DXA scans at the aged 17 research clinic.

### Overall Study Design

The DXA images were performed by trained technicians using a Lunar Prodigy (GE Healthcare, Madison, WI) and were obtained in a standard supine manner. The scans from age 9 and age 15 were combined, then randomly split into a training, a validation and a test set. A similar automated system for three-dimension images of the spine based on magnetic resonance imaging (MRI) scans has already been developed [17]. We planned to modify this system to allow automatic collection of data on spinal scoliosis from total body DXA scans for future research purposes. This modification process was planned to have two stages: (1) development of a new software algorithm to extract the required features and classify spinal images based on a subset of anonymised DXA images from ALSPAC; and (2) validation of the software on a further dataset of anonymised images from ALSPAC.

### Development of the New Software Algorithm

**Fig. 1:**
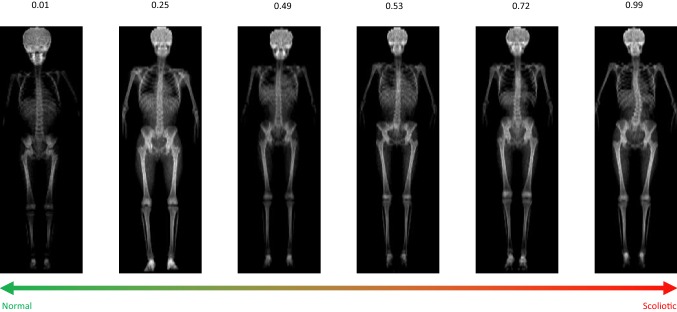
Scoliosis ‘suspiciousness’ scores produced by the automated method. A score of 0 indicates low suspiciousness of scoliosis, and a score of 1 high suspiciousness

As previously reported from the computer science perspective [18], using the training set, all images were standardised to the same height without modifying the aspect ratio (isotropic scaling using the SpineNet software). Segmentations of specific body parts were obtained via simple heuristics by expecting the participants to have two legs, a pelvis, a spine and a head, which are then used to help train the model understand spinal anatomy. Using these segmentations, the first stage of the model was then trained to produce the mid-spine maps—that is a heatmap of which pixels are the most likely to be the middle of the spine. Then, the second stage of the model was trained against the manual classifier of scoliosis/no scoliosis based on the previously validated manual cut-off [9]. The input of the first stage was a DXA image while the input to the second stage was the DXA image and its corresponding mid-spine map. Accuracy of the model was then improved through modifications to labels, maps, and by training against different manual classifications of scoliosis (none, scoliosis with a curve sized 6° to 10°, and scoliosis with a curve sized > 10°) followed by summing these scoliotic classes into one. Each classification produced by the automated model comes with a score—the so-called ‘suspiciousness score’. This score ranges from 0 (normal) to 1 (scoliosis)—see Fig. 1.

### Validation of the Automated Model

**Fig. 2:**
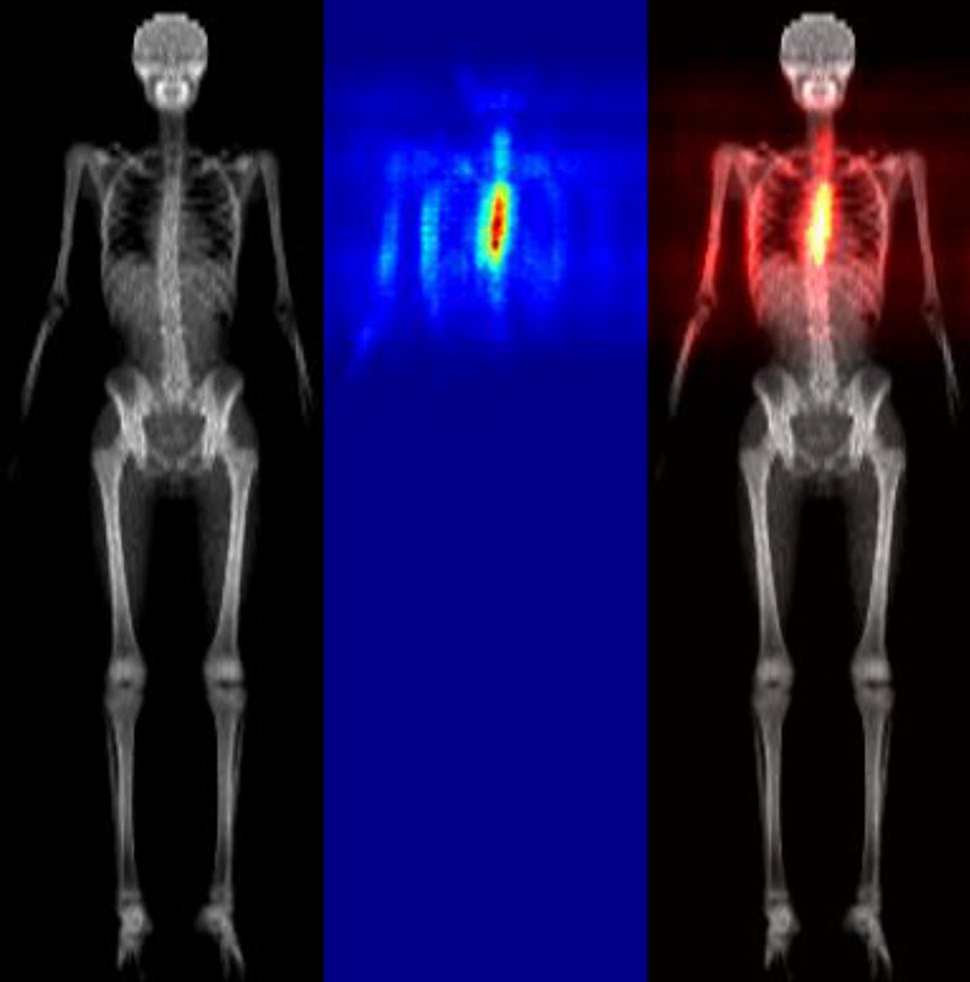
Heatmaps produced by the automation indicating the site of the total body DXA scan that contributed to the decision that scoliosis was present

As before, using the test set, the images were standardised to the same height without modifying the aspect ratio. The trained model was then used to produce a single scoliosis score per test image. To interrogate the model to identify ‘how’ it was making the decisions, heatmaps were produced—see Fig. 2. The brighter pixels in the heatmap are the pixels that contribute the most to the scoliosis prediction.

### Identification of Cut-Off Point for Binary Classification

Scans with evidence of body positioning error based on a score of > 0.5 were excluded. The scoliosis suspiciousness score was reduced to a binary output of scoliosis (yes/no). Different cut-offs of the suspiciousness score were assessed for validity by comparison with the manual DSM annotation on the age 9 and age 15 data using values for sensitivity, specificity and area under the receiver operator curve (AUC). In addition, to estimate the positive predictive value (PPV) and negative predictive value (NPV) of a specific cut-off, the relevant sensitivity and specificity were projected onto a hypothetical population of 10,000 using the known prevalence of 5.9% [9]. The hypothetical population was also used to calculate the percentage of individuals who would be identified (that is, predicted) as having scoliosis according to a given cut-off. A final cut-off (the final model) was chosen by consensus of the study authors based on a high AUC, and optimisation of the sensitivity and PPV.

### Reliability of Final Automated Model

Since identification of spinal curves is influenced by positioning of people on the DXA scanner bed, evaluation of reliability requires assessment of repeat scans in the same individual, with repositioning in-between. Therefore, the automation was also run on a subset of repeat DXA scans carried out 2–6 weeks later in 284 participants from the aged 15 research clinic. To assess reliability, an unweighted Cohen’s kappa statistic was calculated, with standard definitions of categories of agreement [19].

### Assessment of Discrepancies

Using the final cut-off model, where discrepancies between the automated model and manual annotations (DSM) occurred, a random selection of images were reviewed by clinicians (JF and IH who had not previously reviewed these images, and EC who developed the original manual DSM) to identify if the manual annotations were correct or incorrect. All were unaware of the automated prediction, and all read the scans without knowledge of each other’s interpretation. In addition, the automated model was run on the age 17 scans for the discrepancies to identify if the images were still classified as scoliosis by the model, and these age 17 scans were also reviewed by the clinicians.

### Validity of Final Automated Model

The final model was then run on the non-annotated age 17 scans. Percentages were calculated for prevalence of scoliosis. *χ*^2^ tests were used to assess associations between gender and the presence of scoliosis identified by the automated model. Previously, independent associations were identified in ALSPAC between body composition [10] and scoliosis identified by the manual DSM. To assess clinical validity of the automated model, *χ*^2^ tests were used to assess if similar associations were seen between potential predictors (body composition and physical activity) and scoliosis identified by the automated method. For continuous variables such as lean mass unpaired t-tests were used to assess associations with scoliosis.

## Results

The heatmaps of those images with high suspiciousness score for scoliosis consistently highlight specific regions of the spine, indicating these regions contribute the most to the suspiciousness score.

### Identification of the Final Cut-Off Model

After excluding those with body positioning error, different cut-offs of the suspiciousness score were studied: 0.95, 0.98, 0.99, 0.995 and 0.9995—see Table 1. Using a cut-off of suspiciousness score of 0.999, compared with the manual DSM, the automated model has a sensitivity of 86.5%, a specificity of 96.9% and an area under the receiver operator curve value (AUC) of 0.80 (95%CI 0.74 to 0.87)—see Table 2A. This cut-off was then applied to a hypothetical population of 10,000 and has a PPV of 63.6% and an NPV of 99.1%—see Table 2B.

**Table 1:** Identification of the final cut-off point of the continuous suspiciousness score for scoliosis based on the age 15 data after exclusion of those scans with evidence of body positioning error

	Various cut-off levels of the scoliosis suspiciousness score for scoliosis produced by the automation					
	0.95	0.98	0.99	0.995	0.999	0.9995
Using the validation set from ALSPAC						
Sensitivity (%)	94.6	94.6	89.2	89.2	86.5	78.4
Specificity (%)	93.9	94.9	95.2	95.5	96.9	97.8
AUC, 95%CI	0.738 (0.680–0.796)	0.760 0.699–0.820)	0.759 (0.696–0.821)	0.767 (0.704–0.803)	0.804 (0.737–0.871)	0.831 (0.760–0.902)
Applied to a hypothetical population of 10,000						
PPV (%)	49.3	53.8	53.8	55.4	63.6	69.1
NPV (%)	99.6	99.6	99.2	99.3	99.1	98.6
Calculated prevalence (%)	11.3	10.4	9.8	9.5	8.0	6.7

Table shows sensitivity, specificity and AUC calculated from the validation set. The calculated sensitivity and specificity were then applied to a hypothetical population assuming a prevalence of 5.9% to allow calculation of the positive predictive value (PPV), negative predictive value (NPV) and proportion identified with scoliosis by the automated model

**Table 2:** Final model: Automated prediction of scoliosis (suspiciousness score cut off of 0.999) excluding those with body positioning error (suspiciousness score cut-off of 0.5) (A) compared to manual prediction (DSM) based on a test set from within ALSPAC age 9 and age 15 total body DXA scans; and (B) applying the 5.9% prevalence [9], the identified specificity of 96.9% and the identified sensitivity of 86.5% to a hypothetical population of 10,000

Automation	Manual method		
	No scoliosis	Scoliosis	
*(A) Compared to manual prediction in test set*			
No scoliosis	606	5	611
Scoliosis	20	32	52
Total	626	37	663
*(B) Applied to a hypothetical population of 10,000*			
No scoliosis	9118	80	9198
Scoliosis	292	510	802
Total	9410	590	10,000

### Reliability of Final Automated Model

There was almost perfect agreement of identification of those with scoliosis on repeated DXA scans taken 2–6 weeks apart (kappa of 0.90, 95%CI 0.72–1.00).

### Assessment of Discrepancies: Re-assessment of Images by Clinicians

A random sample of 20 of the scans where the manual method and the automated method did not agree were reviewed by three clinicians. Of the scans where the manual method identified no scoliosis, but the automated method did identify scoliosis, 55.6% were re-classified as having scoliosis (in agreement with the automated model) by all three clinicians, suggesting the manual annotation was incorrect in these cases. Similarly, of the scans where the manual method identified scoliosis, but the automated method did not, 60.0% were re-classified as not having scoliosis (in agreement with the automated model) by all three clinicians, suggesting the manual annotation was incorrect in these cases. There was therefore no clear pattern or direction of judgement by the automation. There was no agreement for the remaining discrepant scans as to whether scoliosis was present or not due to the small size of spinal abnormality.

### Assessment of Discrepancies: Comparison with Automated Model Prediction on Age 17 Data

The automated model was run on the age 17 images for those randomly selected discrepant scans described above. For 82.0% of participants, the automated model classified their spines the same at age 15 and age 17, thereby increasing the confidence that the model output is valid.

### Description of Scoliosis Identified by the Automated Model in ALSPAC at Age 17

The descriptive statistics of those with and without scoliosis at age 17 identified by the final automated model is shown in Table 3. As expected, scoliosis was more common in females, but no association was seen with socio-economic status or ethnicity. Similar to previous literature, those with scoliosis at age 17 had lower BMI at age 15. As in previous work by our group [10], those with scoliosis at age 17 had lower total body lean mass.

**Table 3:** Descriptive statistics of those participants from ALSPAC identified by the final automated model with and without scoliosis at age 17, with comparisons by Chi-squared statistics or unpaired t-tests as appropriate

	No scoliosis *N* = 3235	Scoliosis *N* = 449	*P* value for difference
	*N* (%)	*N* (%)	
*Gender*			< 0.001
Male	1526 (91.8)	136 (8.2)	
Female	1709 (84.5)	313 (15.5)	
*Ethnicity*			0.939
White	2783 (87.9)	382 (12.1)	
Non-white	119 (88.2)	16 (11.9)	
*Maternal education*			0.343
Level 1 (none or CSE only)	322 (85.2)	56 (14.8)	
Level 2 (vocational)	219 (90.5)	23 (9.5)	
Level 3 (O levels)	1002 (88.1)	136 (12.0)	
Level 4 (A levels)	824 (87.9)	113 (12.1)	
Level 5 (°)	576 (88.6)	74 (11.4)	
*BMI categories at age 17*			< 0.001
< 18.5	246 (78.6)	67 (21.4)	
18.5–24.9	2193 (86.9)	331 (13.1)	
25.0–29.9	536 (93.1)	40 (6.9)	
≥ 30	244 (95.7)	11 (4.3)	

	Mean (SD)	Mean (SD)	*P* value for difference
Total body lean mass at age 15 (kg)	43.5 (8.4)	39.9 (7.1)	< 0.001

*BMI* body mass index

## Discussion

We have developed a fully automated method of identification of scoliosis from total body DXA scans for research purposes. The final model has good reliability, accuracy, sensitivity, specificity and AUC. Those identified with scoliosis using this method have similar associations with gender, socio-economic status, ethnicity, BMI and lean mass as the known epidemiology of this condition [9, 10]. Disagreement between the automated model and the manual annotation is likely to be explained by errors with the original manual annotation in at least half the cases. Now we are confident the automated model is valid, we are working on training the model to measure size of spinal curve, to allow future research into the predictors of curve size progression.

The benefits of our fully automated model compared to manual annotation of DXA scans is the vast reduction in time required to look at each spinal image, with the consequent large reduction in financial costs. To run the automation on all 12,000 DXA scans from ALSPAC took approximately 5 min. This has resulted in the first feasible and low-radiation process for identification of spinal curves in large populations for research purposes. Other no-radiation techniques are available such as EOS machines, but their use is limited by lack of availability. It is increasingly difficult to justify regular conventional spinal radiography because of the radiation risks, especially to adolescent females who may have an increased risk of breast and uterine carcinoma with increased radiation exposure [20].

The model is not perfect. The sensitivity, specificity and NPV are high, but PPV is low. This, combined with the estimated percentage with scoliosis identified by the model of 8.0%, suggests the model identifies more spinal curves than traditional manual methods. However, it is increasingly recognised that spinal curvature in humans is a continuum, and it is possible our automated method identified more of the small curves than manual methods. Most previous population-based studies of prevalence of scoliosis are based on the Adams forward bending test, and it is recognised that this clinical assessment will miss small curves. It is therefore possible our automated method is correctly identifying a higher prevalence of spinal curves. This could be important, as the current paradigm of using a cut-off of spinal curvature of ≥ 10 ° on standing radiographs [21] carries the implication that lesser curves are not pathological and are ‘normal variants’ [22]. However, previous work by our group has shown that small curves are associated with future back pain and time off school/work [7].

Alternatively, our automated method may be identifying false-positives, but we think this is less likely given that our results are similar to the known epidemiology of scoliosis. The intended purpose of this automated method of scoliosis identification from total body DXA scans is for exploitation of large research datasets. In UK Biobank for example, there will be 100,000 total body DXA scans which will not be able to be analysed for spinal curvature manually because of the enormous time commitment. Our automated method, despite the potential for a proportion of false positives, will allow exploitation of this unique resource, sacrificing some precision for a vast reduction in time required for analysis. Another limitation of this study is that we were unable to confirm that those identified with scoliosis by the automated method were true cases, due to ethical issues regarding over-exposing otherwise normal individuals from ALSPAC to substantial levels of ionising radiation. As previously discussed in the paper describing the validation of the manual method [9], DXA scans are performed in the supine position, which unsurprisingly results in an under-estimation of curve size by approximately 10° in the ALSPAC cohort, similar to other authors [23]. Also as previously published [9], analysis of the supine DXA imaging identifies a higher prevalence of double or triple curves, perhaps explained that without clinical examination we are unable to distinguish compensatory curves that are correctable. However, using a binary cut-off to categorise scans into scoliosis or no scoliosis reduced the impact of this potential limitation.

A final limitation is that both the manual method and the automation described in this paper have been developed on DXA scans performed on a Lunar Prodigy machine produced by GE Healthcare. Other DXA manufacturers are available, (machines produced by GE Healthcare and Hologic comprise the majority), and it is currently unknown how our automation will perform on such images, although we are currently in the process of testing it on Hologic images and outputs are encouraging [un-published data]. However, the intended use of our automation is for research purposes in population-based cohort studies where the serial images are taken on the same machines. We do not intend to use our automation on repeat scans in individuals taken on machines by different manufacturers.

We are now in a position to use this fully automated method to insert the scoliosis phenotype into population-based research cohorts with total body DXA scans around the globe. This will facilitate well-powered studies of the risk factors for initiation of spinal curves, and is likely to produce a step-change in our understanding of this little-researched disease. With future automation development we will also be in the position to study the risk factors for curve progression.
