# Correction to: Identifying Scoliosis in Population‑Based Cohorts: Automation of a Validated Method Based on Total Body Dual Energy X‑ray Absorptiometry Scans

**DOI:** 10.1007/s00223-020-00690-7

**Published:** 2020-04-18

**Authors:** Amir Jamaludin, Jeremy Fairbank, Ian Harding, Timor Kadir, Tim J. Peters, Andrew Zisserman, Emma M. Clark

**Affiliations:** 1 Department of Engineering Science, University of Oxford, Oxford, UK; 2 Nuffield Department of Orthopaedics, Rheumatology and Musculoskeletal Science, University of Oxford, Oxford, UK; 3 North Bristol NHS Trust, Bristol, UK; 4 Optellum Ltd, Oxford, UK; 5 Musculoskeletal Research Unit, Bristol Medical School, University of Bristol, Bristol, UK

## Correction to: Calcified Tissue International (2020) 106:378–385 https://doi.org/10.1007/s00223-019-00651-9

In the original version of the article, the co-author would like to add to the acknowledgements section to highlight their funding stream (EPSRC). The revised acknowledgements is given below.

Orcid number of last author Emma M. Clark inadvertently associated with the first author Amir Jamaludin in the original publication. This has been corrected in this erratum.

### Acknowledgements

We are extremely grateful to all the families who took part in this study, the midwives for their help in recruiting them, and the whole ALSPAC team, which includes interviewers, computer and laboratory technicians, clerical workers, research scientists, volunteers, managers, receptionists, and nurses. The UK Medical Research Council and Wellcome Trust (Grant Ref: 102215/2/13/2) and the University of Bristol provide core support for ALSPAC. A comprehensive list of grants funding is available on the ALSPAC website https://www.bristol.ac.uk/alspac/external/documents/grant-acknowledgements.pdf. This research was specifically funded by the British Scoliosis Research Foundation. This publication is the work of the authors, and EC will serve as guarantor for the contents of this paper, which do not reflect the views of the ALSPAC executive.

Funding for authors based at the University of Oxford was via an EPSRC Programme Grant Seebibyte (EP/M013774/1).

